# Taxonomic Characterization, Whole-Genome Sequencing, and Cosmetic Potential of *Lysinibacillus* sp. JNUCC 51 Isolated from Baengnokdam Crater Lake, Mt. Halla

**DOI:** 10.3390/microorganisms13122786

**Published:** 2025-12-07

**Authors:** Ji-Hyun Kim, Xuhui Liang, Mi-Na Kim, Chang-Gu Hyun

**Affiliations:** Department of Chemistry and Cosmetics, Jeju Inside Agency and Cosmetic Science Center, Jeju National University, Jeju 63243, Republic of Korea; y134g3837@naver.com (J.-H.K.); lxh03036@gmail.com (X.L.); mina@jejunu.ac.kr (M.-N.K.)

**Keywords:** *Lysinibacillus* sp. JNUCC 51, Baengnokdam, chemotaxonomy, fatty acid composition, anti-inflammation, melanin inhibition, cosmetics

## Abstract

A novel bacterial strain, *Lysinibacillus* sp. JNUCC 51, was isolated from volcanic soil collected at Baengnokdam Crater Lake, Mt. Halla, Jeju Island, Republic of Korea. Phylogenetic, ANI (88.76%), and dDDH (70.4%) analyses indicated that the strain represents a distinct genomic lineage closely related to *L. xylanilyticus*. The complete genome (5.12 Mb; 37% G+C) encoded 4912 genes, including ten biosynthetic gene clusters (NRPS, β-lactone, RiPP, terpene, and T3PKS types), suggesting strong metabolic versatility. Cells were Gram-positive rods (1.5–3.0 × 0.5–0.7 µm) growing at pH 4.0–9.0 and up to 5% NaCl. Chemotaxonomic profiles revealed iso-C_15_:_0_, iso-C_17_:_0_, and iso-C_16_:_0_ as dominant fatty acids; MK-6/MK-7 as major quinones; and phosphatidylethanolamine, phosphatidylglycerol, diphosphatidylglycerol, and phosphatidylcholine as main polar lipids. Bioactivity-guided fractionation of the culture extract led to the isolation of Diolmycin A2 (phenolic polyketide) and maculosin (diketopiperazine), both exhibiting anti-inflammatory and melanogenesis-inhibitory effects consistent with their PKS/NRPS gene clusters. The culture broth suppressed nitric oxide production in LPS-stimulated RAW 264.7 macrophages and reduced melanin synthesis in α-MSH–induced B16F10 melanocytes. A human patch test (5% extract) confirmed dermatological safety. Overall, *Lysinibacillus* sp. JNUCC 51 is a volcanic-origin bacterium producing structurally diverse bioactive metabolites with promising postbiotic and cosmeceutical potential, particularly for skin inflammation and pigmentation control.

## 1. Introduction

Jeju Island, located off the southern coast of the Korean Peninsula, is a volcanic island characterized by diverse geological formations and distinct microclimates that have fostered the evolution of unique microbial ecosystems. Among its various volcanic landscapes, Baengnokdam Crater Lake, situated at the summit of Mt. Halla (approximately 1850 m above sea level), represents one of the most pristine and ecologically isolated environments in Korea. The crater is formed on basaltic–trachytic lava deposits and is subject to strong ultraviolet radiation, seasonal temperature fluctuations, and nutrient limitation [1,2,3]. Such physicochemical constraints have shaped a distinctive extremophilic microbial community, wherein microorganisms have evolved specialized metabolic strategies to adapt to low-temperature, oligotrophic, and mineral-rich conditions. These volcanic ecosystems are considered promising biotechnological reservoirs for discovering novel microbial species and bioactive compounds [4,5,6].

Microorganisms from extreme habitats such as volcanic soils, polar regions, or deep-sea sediments have been recognized as valuable sources of unique secondary metabolites with potential applications in medicine, cosmetics, and biotechnology [7,8]. In particular, extremophilic bacteria often produce structurally diverse metabolites involved in stress tolerance and cell protection, including antioxidants, anti-inflammatory agents, and melanin biosynthesis inhibitors [9,10]. These properties align with growing industrial interest in cosmeceutical and dermatological agents derived from natural microorganisms. Previous studies have demonstrated that cold-adapted bacteria can secrete enzymes and bioactive molecules that maintain stability and activity under low-temperature conditions, making them suitable for eco-friendly industrial and pharmaceutical applications. Therefore, the microbial diversity in Baengnokdam’s volcanic soil provides a valuable opportunity to explore novel biogenic materials with biofunctional properties.

Among the diverse bacterial taxa inhabiting volcanic and terrestrial ecosystems, the genus *Lysinibacillus* (family Bacillaceae) has recently gained attention for its broad metabolic versatility and ecological significance. Members of this genus are Gram-positive, endospore-forming, and catalase-positive rods commonly isolated from soil, sediments, and plant-associated environments [11,12]. *Lysinibacillus* species are known to produce a variety of bioactive compounds, including antimicrobial peptides, extracellular enzymes, and anti-inflammatory metabolites [13,14]. For example, *Lysinibacillus macroides* and *L. zambalensis* have been reported to exhibit antioxidant and anti-inflammatory properties [15,16], while *L. xylanilyticus* and *L. sphaericus* are recognized for their ability to degrade polysaccharides and xenobiotic compounds [16,17]. Moreover, certain *Lysinibacillus* strains produce surface-active biomolecules and skin-beneficial metabolites, indicating potential applications in cosmetic formulations and bioremediation [18,19]. The genus is also distinguished by a unique menaquinone composition (predominantly MK-7) and the ability to generate thermostable and pH-resistant enzymes, underscoring its industrial value in enzyme engineering and natural product development [12].

In the present study, we isolated a *Lysinibacillus*-like strain, designated JNUCC 51, from soil collected near Baengnokdam Crater Lake, and investigated its morphological, physiological, and biochemical characteristics. The culture filtrate and solvent-extracted fractions of strain JNUCC 51 were further evaluated for anti-inflammatory and skin-whitening activities using cell-based assays. The extracts inhibited nitric oxide (NO) production in LPS-induced RAW 264.7 macrophages and suppressed melanin biosynthesis in B16F10 melanocytes, suggesting that JNUCC 51 produces bioactive metabolites with potential cosmeceutical applications. These findings highlight the potential of Baengnokdam-derived *Lysinibacillus* strains as natural resources for developing functional ingredients in dermatological and skincare industries.

To gain deeper insight into the genetic and metabolic potential of strain JNUCC 51, whole-genome sequencing and comparative bioinformatic analyses were performed. The genome features—including total length, GC content, and the number of protein-coding and RNA genes—were analyzed, and secondary metabolite biosynthetic gene clusters (BGCs) were predicted using the antiSMASH v8.0.4 platform [20]. The genome was found to encode gene clusters related to polyketide, nonribosomal peptide, and terpenoid biosynthesis, indicating a strong genetic basis for the production of diverse bioactive compounds. These genomic characteristics correspond well with the observed biological activities and support the hypothesis that strain JNUCC 51 represents a promising microbial resource for industrial and biomedical applications.

Collectively, this study provides a comprehensive analysis of a Baengnokdam-derived *Lysinibacillus* strain, integrating phenotypic, biochemical, and genomic approaches with an evaluation of its biological functions. The results contribute to expanding the current understanding of the microbial diversity within volcanic ecosystems of Jeju Island and underline the scientific and biotechnological significance of *Lysinibacillus* spp. As producers of biofunctional natural products.

## 2. Materials and Methods

### 2.1. Chemicals and Reagents

Luria–Bertani (LB) medium was purchased from BD Difco (Becton, Dickinson and Company, Franklin Lakes, NJ, USA). Griess reagent, α-melanocyte-stimulating hormone (α-MSH), sodium hydroxide (NaOH), arbutin, L-DOPA, phosphate-buffered saline (PBS), and protease inhibitor cocktail were obtained from Sigma-Aldrich (St. Louis, MO, USA). Lipopolysaccharides (LPS) from *Escherichia coli* were purchased from VWR (Radnor, PA, USA), and L-NIL was obtained from Cayman Chemical (Ann Arbor, MI, USA). Dimethyl sulfoxide (DMSO) and 3-(4,5-dimethylthiazol-2-yl)-2,5-diphenyltetrazolium bromide (MTT) were purchased from Biosesang (Seongnam, Gyeonggi-do, Republic of Korea). Dulbecco’s Modified Eagle’s Medium (DMEM) and penicillin–streptomycin (1%) were purchased from Thermo Fisher Scientific (Waltham, MA, USA), and fetal bovine serum (FBS) was obtained from Merck Millipore (Burlington, MA, USA). All chemicals and reagents used in this study were of analytical grade or higher purity.

### 2.2. Isolation and Cultivation of the Strain

*Lysinibacillus* sp. JNUCC 51 was isolated from volcanic soil collected at Baengnokdam Crater Lake (33.3611° N, 126.5356° E) on Mt. Halla, Jeju Island, Republic of Korea, in September 2019. A 1.0 g soil sample was suspended in 20 mL of sterile physiological saline and vigorously vortexed to disperse microbial cells. After allowing the soil particles to settle for 30 min, 100 µL of the supernatant was serially diluted (10^−5^–10^−9^) and spread onto Luria–Bertani (LB) agar plates. After 3–4 successive streakings, a pure colony designated JNUCC 51 was obtained and maintained on LB agar. For long-term preservation, the culture was mixed with 20% (*v*/*v*) glycerol and stored at −80 °C. The isolate was routinely cultured in LB broth or agar medium at 30 °C under aerobic conditions, and cells harvested during the late exponential phase were used for genomic DNA extraction.

### 2.3. Extraction and Fractionation of Bacterial Culture

A single colony of *Lysinibacillus* sp. JNUCC 51 was pre-cultured in 5 mL of LB broth at 30 °C for 24 h with shaking (180 rpm). The pre-culture (1 mL) was inoculated into four 500 mL LB broth flasks and incubated at 30 °C for 3 days under the same conditions. After cultivation, the combined culture broths were mixed with an equal volume (2 L) of ethanol and stirred at room temperature for 2 h to obtain the ethanol extract. The resulting mixture was filtered through filter paper (Whatman No. 1), and the filtrate was concentrated under reduced pressure using a rotary vacuum evaporator (Buchi R-210, BÜCHI Labortechnik AG, Flawil, Switzerland) at below 40 °C. The concentrated residue yielded 66.3 g of 80% ethanol extract (JNUCC 51E). A portion of the extract (20.0 g) was suspended in 1 L of distilled water and partitioned three times with an equal volume of ethyl acetate to produce the ethyl acetate fraction (JNUCC 51EA, 600 mg). All extracts and fractions were evaporated to dryness and stored at −20 °C until further use.

### 2.4. Cell Culture and Viability Assay

Murine macrophage RAW264.7 cells were obtained from the Korean Cell Line Bank (KCLB, Seoul, Republic of Korea), and murine melanoma B16F10 cells were purchased from the American Type Culture Collection (ATCC, Manassas, VA, USA).

RAW264.7 cells were cultured in Dulbecco’s Modified Eagle’s Medium (DMEM; Merck Millipore, Burlington, MA, USA) supplemented with 10% fetal bovine serum (FBS; Merck Millipore, MA, USA) and 1% penicillin–streptomycin (Thermo Fisher Scientific, Waltham, MA, USA) at 37 °C in a humidified incubator containing 5% CO_2_. The cells were sub-cultured every two days to maintain exponential growth.

For the cell viability assay, RAW264.7 cells were seeded in 24-well plates at a density of 1.5 × 10^5^ cells/well and treated with various concentrations of the test samples for 24 h. After treatment, MTT solution (0.2 mg/mL) was added and incubated for 3 h at 37 °C. The medium was then discarded, and 800 µL of DMSO was added to dissolve the formazan crystals. Absorbance was measured at 570 nm using a microplate reader (Epoch, Agilent BioTek, Winooski, VT, USA).

Similarly, B16F10 cells were cultured in DMEM supplemented with 10% FBS and 1% penicillin–streptomycin at 37 °C in 5% CO_2_, and sub-cultured every three days. To evaluate cytotoxicity, B16F10 cells were seeded at a density of 8.0 × 10^3^ cells/well in 24-well plates and incubated for 24 h. Cells were treated with varying concentrations of each sample (25–200 µg/mL) for 72 h, followed by the addition of MTT solution (0.2 mg/mL in PBS) and further incubation for 3 h at 37 °C. After removing the medium, 400 µL of DMSO was added to each well to dissolve the formazan crystals. Absorbance was measured at 570 nm using a microplate reader (Epoch, BioTek Instruments, Winooski, VT, USA). Cell viability (%) was expressed relative to untreated control cells.

### 2.5. Determination of Melanin Content

The inhibitory effects of JNUCC 51E and JNUCC 51EA on melanin synthesis were evaluated using B16F10 cells stimulated with α-MSH (100 nM). Cells were seeded in 60 mm culture dishes at 7.0 × 10^4^ cells/well and incubated for 24 h. Various concentrations of the test samples were added along with α-MSH and incubated for 72 h. After treatment, cells were washed twice with 1× PBS and lysed using RIPA buffer containing 1% protease inhibitor cocktail on ice for 20 min. The lysate was collected using a cell scraper, transferred to 1.5 mL microtubes, vortexed for 30 s, and centrifuged at 15,000 rpm for 20 min at −8 °C. The supernatant was removed, and the pellet was dissolved in 250 µL of 1 N NaOH containing 10% DMSO and incubated at 80 °C for 10 min to solubilize melanin. Aliquots (50 µL) of each sample were transferred to a 96-well plate, and absorbance was measured at 405 nm using a microplate reader (Epoch, BioTek Instruments, Winooski, VT, USA). Arbutin (200 µg/mL) was used as a positive control.

### 2.6. Tyrosinase Activity Assay

Tyrosinase activity was determined using the supernatant obtained from the melanin content assay. Protein concentration in each sample was quantified using a BCA Protein Assay Kit (Thermo Fisher Scientific, USA). Each reaction was performed by adding 20 µL of the sample and 80 µL of freshly prepared L-DOPA solution (2 mg/mL in DW) to a 96-well plate, followed by incubation at 37 °C for 1 h. After incubation, absorbance was measured at 450 nm using the same microplate reader. Tyrosinase activity (%) was calculated relative to untreated control samples.

### 2.7. Nitrite Production Assay

Nitric oxide (NO) production was determined by measuring nitrite accumulation in the culture medium using the Griess reaction. RAW264.7 macrophages were seeded in 24-well plates and pre-incubated overnight under standard culture conditions (37 °C, 5% CO_2_). Cells were then stimulated with lipopolysaccharide (LPS; 1 µg/mL) in the absence or presence of the selective iNOS inhibitor L-N^6^-(1-iminoethyl)-lysine (L-NIL; 40 µM). Test samples were co-treated with LPS at various concentrations, with the highest treatment corresponding to 5% (*v*/*v*) of the stock solution. After 24 h incubation at 37 °C in a humidified 5% CO_2_ incubator, the culture supernatants were collected for NO quantification.

Aliquots (100 µL) of each supernatant were transferred to a 96-well plate, and 100 µL of Griess reagent (1% sulfanilamide and 0.1% N-(1-naphthyl)-ethylenediamine dihydrochloride in 2.5% phosphoric acid) was added to each well. After a 10 min reaction at room temperature, the absorbance was measured at 540 nm using a microplate reader (Epoch, Agilent BioTek, USA). Nitrite concentrations were calculated from a standard curve generated with sodium nitrite (NaNO_2_), and results were expressed as µM NO equivalent. All measurements were performed in triplicate.

### 2.8. Genome Sequencing, Assembly, and Annotation

#### 2.8.1. Genomic DNA Extraction and Sequencing

High-molecular-weight genomic DNA (gDNA) was extracted using a commercial purification kit with RNase treatment [21]. A SMRTbell library was prepared and sequenced on the PacBio RS II platform (Pacific Biosciences, Menlo Park, CA, USA) according to the manufacturer’s protocol [22]. After quality control and adapter trimming, a total of 140,500 subreads amounting to 1.44 Gb were obtained, with a mean subread length of 10,281 bp and an N50 value of 14,761 bp. In parallel, an Illumina paired-end library (2 × 150 bp) was constructed and sequenced on an Illumina platform [23]. After trimming and filtering to remove low-quality reads and adapters, 5,846,834 clean reads (total = 882.9 Mb, GC content = 36.79%) were retained for hybrid assembly and downstream analyses [24].

#### 2.8.2. Read Processing and Genome Size Estimation

Filtered Illumina reads were analyzed using Jellyfish v2.2.10 [25] and GenomeScope 2.0 [26] for 21-mer distribution modeling. The estimated genome size was 5.11 Mb (coverage = 34×; heterozygosity = 0.026).

#### 2.8.3. De Novo Assembly and Polishing

PacBio long reads were assembled using HGAP3 implemented in SMRT Portal v2.3 [22], producing a single circular contig. Short-read polishing was conducted with Pilon v1.21 [27], mapping the filtered Illumina reads to the draft assembly for base correction. The final genome comprised one circular chromosome of 5,122,675 bp with 36.87% GC content and an average long-read coverage of ~221×.

#### 2.8.4. Assembly Validation

Illumina reads were mapped to the polished genome, yielding 99.98% mapped reads and 100% coverage [28]. Genome completeness was assessed using BUSCO v3.0 [29], showing 98.65% completeness. Taxonomic placement was confirmed using BLASTN v2.7.1+ [30] against the NCBI nt database.

#### 2.8.5. Genome Annotation and Biosynthetic Gene Cluster Analysis

Functional annotation was performed using Prokka v1.12b [31], identifying 4782 CDSs, 107 tRNAs, and 43 rRNAs. Circular genome visualization (CDSs, tRNA/rRNA loci, GC content, GC skew) was generated from the annotated GenBank file. Secondary metabolite biosynthetic gene clusters were predicted using antiSMASH v8.0 [32].

#### 2.8.6. Data Records and Availability

Sequencing reads and the assembled genome will be deposited in the NCBI SRA and GenBank under a BioProject accession to be assigned [21].

Deliverables included PacBio (.bax/.bas) and Illumina (FASTQ) data, assembled FASTA/GBK/GFF3 files, annotation tables, CDS (.ffn/.faa) datasets, and quality assessment reports (BUSCO, coverage, methylation motif).

### 2.9. Physiological Characterization

Strain JNUCC 51 was physiologically characterized by determining its growth response to temperature, pH, and NaCl concentration. For temperature-dependent growth, the strain was inoculated into 3 mL of sterile LB broth (Difco, Atlanta, GA, USA) in 15 mL conical tubes and incubated at 10, 20, 30, 37, and 42 °C under agitation at 200 rpm for 24 h. The effect of pH on growth was examined in LB broth adjusted to pH 4.0, 5.0, 6.0, 7.0, 8.0, 9.0, and 10.0 using 1 N HCl or 1 N NaOH after calibration at pH 7.0 with a standard buffer solution. For each condition, 3 mL of pH-adjusted medium was dispensed into 15 mL conical tubes, and 30 µL of an overnight culture of strain JNUCC 51 was inoculated. The cultures were incubated at 30 °C and 200 rpm for 24 h. Similarly, salt tolerance was determined by cultivating the strain in LB broth supplemented with different NaCl concentrations. A 30% (*w*/*v*) NaCl stock solution was prepared and added to achieve final concentrations of 0%, 1%, 3%, 5%, 7%, and 10% (*w*/*v*). Each 15 mL tube contained 3 mL of NaCl-supplemented medium, and 30 µL of the inoculum was added. All cultures were incubated at 30 °C and 200 rpm for 24 h. After incubation, 200 µL of each culture from the temperature, pH, and NaCl tests was transferred into a 96-well microplate in triplicate, and growth was measured spectrophotometrically at 600 nm (OD_600_) using a microplate reader (Epoch, BioTek Instruments, Winooski, VT, USA).

### 2.10. Chemotaxonomic Analyses

#### 2.10.1. Enzyme Activity Profiling

Enzymatic activities of strain JNUCC 51 were determined using the API ZYM system (bioMérieux, Craponne, France) following the manufacturer’s instructions. Cells were grown on LB agar (Difco, USA) at 30 °C for 24 h and suspended in sterile 0.85% NaCl solution to reach a turbidity equivalent to McFarland standard 5.0. Each cupule of the API ZYM strip was inoculated with 65 µL of the cell suspension and incubated in a humid chamber at 37 °C for 4 h. After incubation, one drop each of reagents ZYM A and ZYM B was added to each well, and color development was evaluated after 5 min. Enzyme activities were scored from 0 (no activity) to 5 (strong activity) according to the color intensity. *Escherichia coli* K-12 and sterile saline were used as positive and negative controls, respectively.

#### 2.10.2. Cellular Fatty Acid Composition

Cellular fatty acid composition was analyzed using the Sherlock Microbial Identification System (MIDI, Newark, DE, USA) according to the TSBA6 protocol. Cells were harvested from LB agar plates incubated at 30 °C for 24 h, corresponding to the late exponential growth phase. The cells (40–50 mg wet weight) were subjected to saponification with 15% NaOH in 50% methanol (1.0 mL) at 100 °C for 30 min, followed by methylation with 6 N HCl in methanol (2.0 mL) at 80 °C for 10 min to produce fatty acid methyl esters (FAMEs). The FAMEs were extracted with hexane/methyl tert-butyl ether (1:1, *v*/*v*) and washed with 1.2% NaOH. Gas chromatography (GC) was carried out on an Agilent 7890B instrument (Agilent Technologies, Santa Clara, CA, USA) equipped with an HP-Ultra 2 capillary column (25 m × 0.2 mm, 0.33 µm film thickness) and a flame ionization detector. Helium was used as the carrier gas at a constant flow rate of 1.0 mL min^−1^. The temperature program followed the standard TSBA6 method. Fatty acids were identified and quantified by comparing the retention times and equivalent chain lengths with those in the MIDI Sherlock TSBA6 database (version 6.2).

#### 2.10.3. Polar Lipid Analysis

Polar lipids were extracted from freeze-dried cells (~200 mg) cultivated in LB broth at 30 °C for 48 h using a chloroform/methanol/water mixture (1:2:0.8, *v*/*v*/*v*), following the modified Bligh and Dyer method. The organic layer was collected, evaporated under nitrogen, and redissolved in chloroform. Two-dimensional thin-layer chromatography (TLC) was performed on silica gel 60 HPTLC plates (Merck, Darmstadt, Germany). The first dimension was developed with chloroform/methanol/water (65:25:4, *v*/*v*/*v*) and the second with chloroform/acetic acid/methanol/water (40:7.5:6:2, *v*/*v*/*v*/*v*). Lipid spots were visualized using 10% phosphomolybdic acid in ethanol for total lipids, 0.2% ninhydrin solution for aminolipids, and the Dittmer–Lester reagent for phospholipids. The lipid profile was compared with reference standards for phosphatidylethanolamine (PE), phosphatidylglycerol (PG), diphosphatidylglycerol (DPG), and phosphatidylcholine (PC).

#### 2.10.4. Isoprenoid Quinone Composition

Isoprenoid quinones were extracted from freeze-dried cells (200–500 mg) cultivated in LB broth at 30 °C for 48 h using hot methanol (60 °C, 10 min) and partitioned with hexane (1:1, *v*/*v*). The hexane fraction was evaporated and further purified by silica gel TLC with hexane/diethyl ether (85:15, *v*/*v*) as the developing solvent. The purified quinone extract was analyzed by high-performance liquid chromatography (HPLC) on a C18 reverse-phase column (150 × 4.6 mm, 5 µm; Agilent, Santa Clara, CA, USA) with methanol/isopropanol (3:2, *v*/*v*) as the mobile phase at a flow rate of 1.0 mL min^−1^. Detection was performed at 270 nm using a photodiode array detector. Quinone species were identified by comparing the retention times and UV spectra with those of authentic menaquinone standards (MK-6 from *Flavobacterium johnsoniae* KCCM 41691 and MK-7 chemical standard from Sigma-Aldrich).

### 2.11. Genome-Based Taxonomic Analysis

Whole-genome-based taxonomic analysis of *Lysinibacillus* sp. JNUCC 51 was performed using the Type (Strain) Genome Server (TYGS; https://tygs.dsmz.de, accessed on 20 August 2025r) [33]. The analysis followed the standardized genome-to-genome distance phylogeny (GBDP) framework for species delineation [33,34]. The uploaded genome was compared with all available type-strain genomes in the TYGS database, and the ten most closely related type strains were selected based on intergenomic distances and 16S rRNA similarity. Digital DNA–DNA hybridization (dDDH) values were computed using the Genome-to-Genome Distance Calculator (GGDC 4.0) embedded in TYGS, employing formulas d0, d4, and d6. Phylogenetic trees were inferred by the balanced minimum-evolution algorithm with 100 pseudo-bootstrap replicates.

The results Indicated that strain JNUCC 51 Is most closely related to *L. xylanilyticus* DSM 23493^T^, exhibiting dDDH values of 70.4%(d0), 67.0%(d4), and 72.1%(d6), and a ΔG + C difference of 0.25%, supporting its recognition as a novel species within the genus *Lysinibacillus*.

To complement the dDDH analysis, pairwise Average Nucleotide Identity (ANI) values were calculated using the CJ Bioscience Genome Analysis Service implementing the OrthoANIu algorithm (https://www.ezbiocloud.net/tools/ani; accessed on 20 August 2025) [35]. The highest ANI value, obtained with *L. xylanilyticus* DSM 23493^T^, remained below the 95–96% species boundary [36], further confirming that JNUCC 51 represents a distinct genomic species.

### 2.12. Scanning Electron Microscopy (SEM)

Cells of strain JNUCC 51 were observed using a high-resolution field emission scanning electron microscope (FE-SEM; Regulus 8100, Hitachi, Tokyo, Japan). For SEM observation, the strain was cultivated on LB agar plates at 30 °C for 24 h. A loopful of cells was gently collected and fixed with 2.5% (*v*/*v*) glutaraldehyde in 0.1 M phosphate buffer (pH 7.2) for 2 h at 4 °C. The fixed samples were washed three times with the same buffer and subsequently dehydrated through a graded ethanol series (30%, 50%, 70%, 90%, and 100%, 10 min each). After critical point drying, the samples were mounted on aluminum stubs, sputter-coated with platinum to a thickness of approximately 10 nm, and examined at an accelerating voltage of 5.0 kV, a working distance of 8.0 mm, and magnifications ranging from ×8000 to ×25,000.

### 2.13. Fermentation, Extraction, and Isolation

The strain *Lysinibacillus* sp. JNUCC51, isolated from volcanic soil collected at Baengnokdam Crater, Mt. Halla, Jeju Island, Republic of Korea, was maintained on LB agar and used for metabolite production in LB broth. A seed culture was prepared by inoculating the strain into 125 mL of LB broth in a 250 mL Erlenmeyer flask and incubating at 30 °C for 48 h under shaking conditions (150 rpm). The resulting culture (5%, *v*/*v*) was transferred into four 5 L Erlenmeyer flasks, each containing 1 L of LB medium, and further incubated aerobically at 30 °C for 4 days. After cultivation, the 5 L culture broth was filtered through 300 mm filter paper (ADVANTEC, Tokyo, Japan) to remove cells and debris. The clear filtrate was extracted three times with ethyl acetate (EtOAc, 4 L × 3), and the combined organic layers were concentrated under reduced pressure to yield a crude extract (400 mg). The EtOAc extract was subjected to silica gel column chromatography using a stepwise chloroform–methanol gradient (300 mL each) to obtain ten fractions (Fr. V1–V10). Fraction V10 was further purified on a silica gel column eluted with chloroform–methanol (20:1, *v*/*v*), yielding Diolmycin A2 (26 mg), while fraction V8, eluted with chloroform–methanol (50:1, *v*/*v*), afforded maculosin (15 mg). The isolated compounds were structurally characterized by ^1^H and ^13^C NMR spectroscopy (500 and 126 MHz, respectively, in methanol-d_4_) and high-resolution electrospray ionization mass spectrometry (HR-ESI-MS).

### 2.14. Human Skin Patch Test

This study was reviewed and approved by the Institutional Review Board (IRB) of Dermapro Co., Ltd. (Approval No. 1-220777-A-N-01-B-DICN25264) in compliance with the ethical principles of the Declaration of Helsinki. All participants provided written informed consent before participation. Thirty-two healthy adults (31 females and 1 male), aged 26–54 years (mean ± SD: 47.78 ± 6.31), who met the inclusion and exclusion criteria were enrolled. The purpose, procedures, and potential risks of the study were fully explained to all subjects prior to testing.

A designated area on the upper back of each subject was cleansed with 70% ethanol, and 20 μL of the 2.5% and 5% (*v*/*v*) culture broth samples were applied under occlusion for 24 h. Skin responses were assessed twice: first, 20 min after patch removal, and again 24 h later. A board-certified dermatologist evaluated local skin reactions following the Personal Care Products Council (PCPC) guidelines using both visual inspection and standardized questionnaires.

If any adverse reactions were observed, appropriate medical care was provided immediately to ensure participant safety. Reaction intensity for the 2.5% and 5% culture broth samples was graded according to a predefined scoring system, and the mean reactivity was calculated. Following PCPC recommendations, any reaction graded +5 was interpreted as possibly allergic rather than irritant; therefore, the maximum grade used in this study was +4.


$$Response\;=\;\frac{\sum \left(Grade\;\times \;No.\;\;of\;Responders\right)}{4\;(Maximum\;Grade)\;\times \;n\;(Total\;Subjects)}\;\times \;100\;\times \;1/2$$

### 2.15. Statistical Analyses

All statistical analyses were performed using IBM SPSS Statistics software (version 20; SPSS Inc., Armonk, NY, USA). Differences among groups were analyzed using one-way analysis of variance (ANOVA) followed by Tukey’s post hoc test. Data are expressed as the mean ± standard deviation (SD) from three independent experiments. For graphical representation, statistical significance was indicated as follows: ^#^ *p* < 0.05 vs. the untreated control group; * *p* < 0.05, ** *p* < 0.01, and *** *p* < 0.001 vs. the LPS-treated or α-MSH–treated group, depending on the experimental design.

## 3. Results

### 3.1. Phylogenetic Analysis

A comparative 16S rRNA gene sequence analysis (1478 bp) was conducted to determine the phylogenetic position of strain JNUCC 51. Sequence similarity searches using the EzBioCloud (https://www.ezbiocloud.net/; accessed on 20 August 2025) and GenBank/EMBL/DDBJ databases (https://www.ncbi.nlm.nih.gov/; accessed on 20 August 2025) indicated that the isolate shared high sequence identities with *Lysinibacillus boronitolerans* NBRC 103108 (NR_114207.1, 99.19%), *L. macroides* LMG 18474 (NR_114920.1, 98.99%), *L. fusiformis* NBRC 15717 (NR_112628.1, 98.44%), and *L. pakistanensis* NCCP-54 (NR_113166.1, 98.64%). Phylogenetic inference was performed using the maximum likelihood (ML) and neighbor-joining (NJ) algorithms implemented in MEGA version 11 [37], with bootstrap resampling based on 1000 replicates to assess the robustness of the tree topology. The resulting phylogenetic tree clearly positioned strain JNUCC 51 within the genus *Lysinibacillus*, belonging to the family Bacillaceae.

### 3.2. Effect of JNUCC 51 Extracts on Melanogenesis in B16F10 Cells

**Figure 1:**
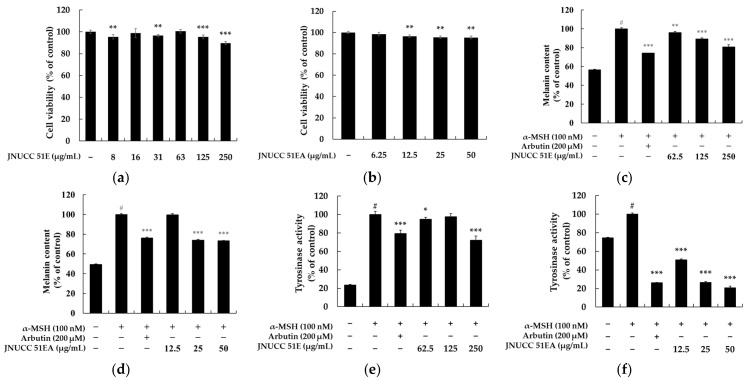
Effects of *Lysinibacillus* sp. JNUCC 51 extracts on cell viability, melanin content, and tyrosinase activity in B16F10 melanoma cells. (**a**,**b**) Cell viability of B16F10 cells treated with various concentrations of JNUCC 51 80% ethanolic extract (JNUCC 51E) and its ethyl acetate fraction (JNUCC 51EA) for 72 h, as determined by the MTT assay. (**c**,**d**) Effects of JNUCC 51E and JNUCC 51EA on melanin synthesis in α-MSH (100 nM)-stimulated B16F10 cells compared with untreated and arbutin-treated (200 μM) controls. (**e**,**f**) Intracellular tyrosinase activity following treatment with JNUCC 51E and JNUCC 51EA under the same conditions. Data are expressed as the mean ± SD of three independent experiments. ^#^ *p* < 0.05 vs. untreated control; * *p* < 0.05, ** *p* < 0.01, and *** *p* < 0.001 vs. α-MSH–treated group.

The effects of the JNUCC 51 extracts on melanogenesis in B16F10 melanoma cells were investigated. To determine the non-cytotoxic concentration range, cell viability was evaluated using the MTT assay after 72 h of treatment with various concentrations of each sample. Concentrations maintaining more than 90% cell viability compared with untreated controls were regarded as non-toxic. The 80% ethanolic extract of JNUCC 51 (JNUCC 51E) did not show cytotoxicity up to 250 μg/mL, while the ethyl acetate fraction (JNUCC 51EA fraction) was non-toxic below 50 μg/mL. Based on these results, subsequent experiments were conducted using the JNUCC 51E at concentrations of 62.5, 125, and 250 μg/mL, and the JNUCC 51EA at 12.5, 25, and 50 μg/mL. The inhibitory effects of the JNUCC 51E and JNUCC 51EA on melanin production were then assessed under non-cytotoxic conditions. B16F10 cells were treated with each sample for 72 h in the presence of α-MSH (100 nM) as a negative control and arbutin (200 μM) as a positive control. The JNUCC 51E significantly reduced melanin content in a dose-dependent manner, showing a 21.5% decrease at 250 μg/mL, while the JNUCC 51EA caused a 26.7% reduction at 50 μg/mL compared with α-MSH-stimulated cells. Furthermore, intracellular tyrosinase activity was measured to clarify the mechanism underlying the inhibitory effects on melanogenesis. The JNUCC 51E reduced tyrosinase activity by 35.5% at 250 μg/mL, and the JNUCC 51EA exhibited a stronger effect, showing a 50.6% decrease at 50 μg/mL relative to the α-MSH control. Arbutin (200 μM) effectively inhibited tyrosinase activity, validating the assay system. Taken together, these results indicate that both the ethanolic extract and its ethyl acetate fraction derived from strain JNUCC 51 suppress melanogenesis in B16F10 cells by attenuating tyrosinase activity, with the JNUCC 51EA displaying greater potency (Figure 1).

### 3.3. Effects of Lysinibacillus sp. JNUCC 51 Broth on Nitrite Production in LPS-Stimulated RAW264.7 Cells

**Figure 2:**
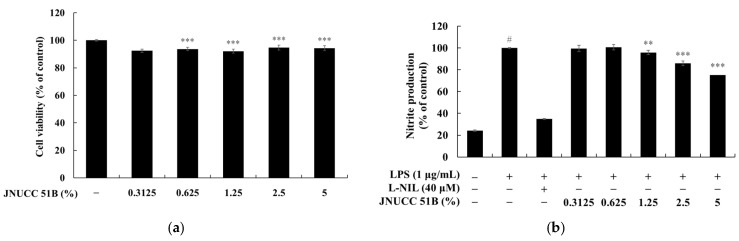
Effects of (**a**) *Lysinibacillus* sp. JNUCC 51 broth (JNUCC 51B) on LPS-induced nitrite production in RAW264.7 macrophages. (**b**) Cells were treated with LPS (1 µg/mL) in the presence or absence of L-NIL (40 µM) and JNUCC 51B (0.3125–5%, *v*/*v*) for 24 h. Nitrite levels were determined using the Griess assay, and values are expressed as the percentage of the LPS-treated group. Data are expressed as the mean ± SD (*n* = 3). ^#^ *p* < 0.05 vs. untreated control; ** *p* < 0.01, *** *p* < 0.001 vs. LPS-treated group.

The inhibitory effect of *Lysinibacillus* sp. JNUCC 51 broth (JNUCC 51B) on nitric oxide (NO) production was assessed in LPS-stimulated RAW264.7 macrophages. As shown in Figure 2, treatment with LPS (1 µg/mL) significantly increased nitrite production by approximately 4.2-fold compared with the untreated control (*p* < 0.001, ^#^), confirming the induction of an inflammatory response. In contrast, treatment with the selective iNOS inhibitor L-NIL (40 µM) markedly suppressed nitrite accumulation by 65.21%, validating the reliability of the experimental system.

Co-treatment with *Lysinibacillus* sp. JNUCC 51 Broth (JNUCC 51B) reduced LPS-induced NO production in a dose-dependent manner. Nitrite levels decreased by 4.18%, 14.24%, and 24.89% at JNUCC 51B concentrations of 1.25%, 2.5%, and 5% (*v*/*v*), respectively, relative to the LPS-treated group (*p* < 0.001, ***). Lower concentrations (0.3125% and 0.625%) showed no statistically significant effect. These results demonstrate that metabolites present in the JNUCC 51B effectively suppress LPS-induced NO production in macrophages, suggesting that JNUCC 51B exerts anti-inflammatory activity through the inhibition of iNOS-mediated nitric oxide synthesis.

### 3.4. Taxonomic Characterization of Lysinibacillus sp. JNUCC 51

Comprehensive taxonomic analyses were conducted to characterize the phenotypic, chemotaxonomic, and physiological traits of strain *Lysinibacillus* sp. JNUCC 51 in comparison with its closest relative, *L. xylanilyticus* DSM 23493ᵀ, based on genome and 16S rRNA gene similarities. Strain JNUCC 51 was capable of growth within a pH range of 4.0–9.0 and tolerated up to 5% (*w*/*v*) NaCl, whereas the reference strain grew between pH 5.0 and 9.0 and up to 3% NaCl. Both strains showed optimal growth at 30 °C, with weaker growth at 20 °C and 42 °C, indicating that JNUCC 51 exhibits slightly broader environmental adaptability than the type strain (Table 1). The API ZYM test revealed positive enzymatic reactions for esterase (C4), acid phosphatase, and naphthol-AS-BI-phosphohydrolase in strain JNUCC 51, while *L. xylanilyticus* additionally showed activities for alkaline phosphatase, leucine arylamidase, β-galactosidase, and α-glucosidase, suggesting minor but consistent biochemical variation (Table 2). Cellular fatty acid analysis showed that branched-chain fatty acids predominated in strain JNUCC 51 (≈85% of total), with iso-C_15:0_ (52.7%), iso-C_17:0_ (11.6%), iso-C_16:0_ (6.9%), and anteiso-C_15:0_ (7.2%) as the major components. This fatty-acid pattern aligns with the genus *Lysinibacillus* but differs slightly from *L. xylanilyticus* in the relative abundance of anteiso-branched species (Table 3). Two-dimensional thin-layer chromatography showed that strain JNUCC 51 contained phosphatidylethanolamine (PE), phosphatidylglycerol (PG), and diphosphatidylglycerol (DPG) as major polar lipids, with additional minor components including phosphatidylcholine (PC), one unidentified phospholipid (PL), and one unidentified lipid (L), whereas *L. xylanilyticus* DSM 23493ᵀ contained only PE, PG, and DPG (Table 4). HPLC analysis of the isoprenoid quinone system revealed menaquinone-7 (MK-7) as the predominant component and a small amount of MK-6. This quinone composition is typical of members of *Lysinibacillus*. Collectively, strain JNUCC 51 shares the general chemotaxonomic characteristics of the genus *Lysinibacillus* but exhibits several differentiating traits from *L. xylanilyticus* DSM 23493ᵀ, particularly in its polar-lipid composition and fatty-acid profile, suggesting that JNUCC 51 may represent a distinct lineage within the genus *Lysinibacillus*, pending further taxonomic confirmation.

### 3.5. Sequencing, Assembly, and Genomic Characteristics

The complete genome of *Lysinibacillus* sp. JNUCC 51 was obtained using a hybrid sequencing approach that combined PacBio RS II long reads and Illumina paired-end short reads. The PacBio dataset comprised 140,500 subreads totaling 1.44 Gb (mean read length 10,281 bp, N50 = 14,761 bp), while the Illumina library generated 8.1 million reads (1.23 Gb) after quality trimming, yielding Q30 > 98%.

De novo assembly was carried out using HGAP3 in SMRT Portal v2.3, and the resulting consensus sequence was polished with Pilon v1.21 using the Illumina data. The final assembly produced a single circular chromosome of 5,122,675 bp with 37% G+C content and an average coverage depth of 221×. No plasmid sequences were detected. Genome completeness evaluated by BUSCO v3.0 (bacteria_odb9 dataset) indicated 98.65% complete and 0.68% missing orthologs, confirming high assembly accuracy.

Automated annotation using Prokka v1.12b predicted a total of 4912 genes, including 4615 protein-coding sequences, 141 pseudogenes, 108 tRNA genes, 43 rRNA genes (15 for 5S, 14 for 16S, and 14 for 23S), and 5 ncRNA genes. The genome architecture is consistent with the genus *Lysinibacillus*, exhibiting a single chromosome without plasmids.

The general features of the *Lysinibacillus* sp. JNUCC 51 genome are summarized in Table 5. The genome (5.12 Mb, 37% GC) is organized as a single circular chromosome with full complements of rRNA and tRNA genes, providing a stable genetic framework for functional and comparative genomic analyses.

### 3.6. Characterization of Lysinibacillus sp. JNUCC 51 as a Novel Strain via dDDH Analysis

Whole-genome relatedness between *Lysinibacillus* sp. JNUCC 51 and type strains of the genus *Lysinibacillus* was assessed using the Genome-to-Genome Distance Calculator (GGDC 4.0) based on the digital DNA–DNA hybridization (dDDH) method. Pairwise dDDH estimates were computed under the recommended distance formulas d_0_ (HSP length/total length), d_4_ (identities/HSP length), and d_6_ (identities/total length) with 100 distance replicates to provide 95% confidence intervals.

The dDDH results (Table 6) revealed that strain JNUCC 51 shared the highest genomic similarity with *Lysinibacillus xylanilyticus* DSM 23493ᵀ, showing 70.4% (d_0_), 67.0% (d_4_), and 72.1% (d_6_) relatedness, with a ΔG + C content difference of 0.25%. These values are near but slightly above the classical 70% threshold for species demarcation, suggesting that JNUCC 51 is closely related to *L. xylanilyticus* but may represent a distinct genomic lineage within the genus.

All other type strains exhibited significantly lower dDDH values (<55% for *L. zambalensis* M3, <48% for *Bacillus decisifrondis* DSM 17725ᵀ, and <30% for the remaining *Lysinibacillus* species), far below the boundary for species-level assignment. The small G + C content variation (≤1%) among related taxa supports the affiliation of JNUCC 51 within the genus *Lysinibacillus*.

Based on the combined evidence from dDDH, genome size (5.12 Mb, 37% GC), and phylogenomic context, *Lysinibacillus* sp. JNUCC 51 is proposed as a novel genomic variant closely allied to *L. xylanilyticus*, pending further phenotypic and biochemical differentiation to validate its taxonomic status.

### 3.7. Characterization of Lysinibacillus sp. JNUCC 51 as a Novel Strain via ANI Analysis

To further validate the genomic relatedness between *Lysinibacillus* sp. JNUCC 51 and its closest relative identified by dDDH analysis, a pairwise average nucleotide identity (ANI) comparison was performed using the OrthoANIu algorithm implemented by CJ Bioscience (Seoul, Republic of Korea). The complete genome of *Lysinibacillus* sp. JNUCC 51 (5,122,440 bp) was compared with that of *L. xylanilyticus* DSM 23493ᵀ (5,049,000 bp).

The OrthoANIu value between the two genomes was 88.76%, based on an average aligned length of 2,315,154 bp, with respective genome coverages of 45.20% (JNUCC 51) and 45.85% (*L. xylanilyticus*). These values are notably below the widely accepted species boundary of 95–96%, which is typically used to delineate prokaryotic species (Table 7).

The relatively low ANI similarity, together with the dDDH values (70.4%, 67.0%, and 72.1% under formulas d_0_–d_6_), indicates that *Lysinibacillus* sp. JNUCC 51 is genetically distinct from *L. xylanilyticus* despite their close phylogenetic relationship. The combination of ANI and dDDH results thus supports the interpretation that strain JNUCC 51 represents a novel genomic lineage within the genus *Lysinibacillus*.

### 3.8. In Silico Prediction of Secondary Metabolites and Biosynthetic Gene Cluster Analysis

The genome of *Lysinibacillus* sp. JNUCC 51 was examined using antiSMASH v8.0 to identify potential biosynthetic gene clusters (BGCs) responsible for secondary metabolite production. A total of ten putative BGCs were detected across the 5.12 Mb genome (Table 8), representing a diverse array of metabolite classes, including nonribosomal peptide synthetase (NRPS), terpene, betalactone, type III polyketide synthase (T3PKS), siderophore, and azole-containing RiPP clusters.

Among these, three clusters exhibited notable similarity to known biosynthetic pathways. The NI-siderophore cluster (Region 1) showed low-confidence similarity to the petrobactin biosynthetic cluster, which is typically associated with iron-chelating activity in Bacillus and *Lysinibacillus* species. The betalactone cluster (Region 4) displayed similarity to the fengycin synthetase complex (NRPS-Type I), a lipopeptide with well-established antimicrobial and surfactant properties. Another NRPS-Type I cluster (Region 7) showed low-confidence similarity to bacillibactin/E/F, suggesting the potential for siderophore-like biosynthesis.

The remaining clusters, including those encoding terpenes, terpene-precursors, and azole-containing RiPPs, lacked significant homology to characterized BGCs, implying that they may encode novel or cryptic secondary metabolites not yet functionally annotated in public databases. The presence of multiple NRPS and terpene clusters indicates that strain JNUCC 51 harbors a substantial biosynthetic capacity for antimicrobial, surface-active, and metal-chelating compounds, consistent with the metabolic diversity commonly observed in *Lysinibacillus* species.

These findings highlight *Lysinibacillus* sp. JNUCC 51 as a promising microbial resource for the discovery of previously uncharacterized bioactive molecules, particularly within its NRPS and betalactone biosynthetic region.

### 3.9. Morphological Features Under Scanning Electron Microscopy

**Figure 3:**
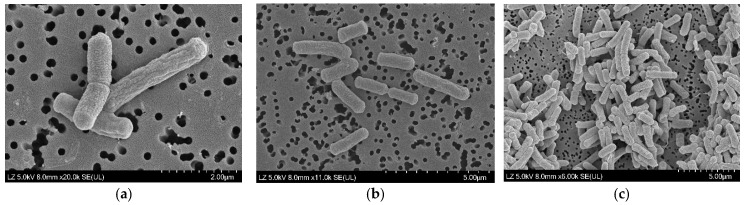
High-resolution field emission scanning electron micrographs of strain *Lysinibacillus* sp. JNUCC 51. (**a**) High-magnification image (×20,000; scale bar = 2 μm) showing detailed surface structures of rod-shaped cells with smooth to slightly wrinkled textures. (**b**) Intermediate-magnification image (×11,000; scale bar = 5 μm) showing individual cells measuring approximately 1.5–3.0 μm in length and 0.5–0.7 μm in width. (**c**) Low-magnification image (×6000; scale bar = 5 μm) showing cell clusters exhibiting rod-shaped morphology.

High-resolution FE-SEM micrographs revealed that cells of strain JNUCC 51 were rod-shaped with rounded ends and measured approximately 0.6–0.9 μm in width and 2.0–3.5 μm in length. The cell surface appeared smooth to slightly wrinkled, and individual cells were often observed as single rods or in short chains, occasionally forming pairs. No flagella or spore-like structures were visible under the tested conditions (Figure 3). These morphological characteristics are consistent with the general features of the genus *Lysinibacillus*.

### 3.10. Isolation and Identification of Secondary Metabolites

Large-scale fermentation of *Lysinibacillus* sp. JNUCC51 in Luria–Bertani (LB) medium yielded approximately 5 L of culture broth, which was extracted with ethyl acetate to afford 400 mg of a crude organic extract. Sequential fractionation of the extract by silica gel column chromatography generated ten fractions (Fr. V1–V10) that exhibited distinct chromatographic profiles. Bioactivity-guided purification, supported by TLC monitoring, led to the isolation of two major metabolites: Diolmycin A2 (26 mg) from fraction V10 and maculosin (15 mg) from fraction V8. Both compounds were obtained as colorless amorphous solids. Their structural identities were determined through comprehensive spectroscopic analyses, including ^1^H and ^13^C NMR spectroscopy and high-resolution electrospray ionization mass spectrometry (HR-ESI-MS) (Figures S1–S5). The spectroscopic data were in full agreement with previously reported literature values [38,39].

For Diolmycin A2, the spectroscopic data were as follows: ^1^H NMR (500 MHz, methanol-d_4_) δ 7.41–7.27 (m, 2H), 7.10–7.07 (m, 2H), 7.01–6.98 (m, 2H), 6.82–6.76 (m, 1H), 6.70–6.64 (m, 2H), 3.90 (s, 1H), 3.59 (d, J = 9.0 Hz, 1H), 3.26–2.64 (m, 5H); ^13^C NMR (126 MHz, methanol-d_4_) δ 157.11, 138.02, 131.80, 131.49, 129.57, 124.66, 122.49, 119.90, 119.56, 116.17, 112.24, 110.96, 108.03, 78.22, 77.55, 40.39, 31.16; HRMS (ESI) *m*/*z* [M − H]^−^ 296.1298 (calcd for C_18_H_19_NO_3_, 296.1292).

For maculosin, the spectroscopic data were as follows: ^1^H NMR (500 MHz, methanol-d_4_) δ 7.04–6.98 (m, 2H), 6.71–6.64 (m, 2H), 4.33 (td, J = 4.8, 1.9 Hz, 1H), 4.02 (ddd, J = 10.9, 6.3, 2.0 Hz, 1H), 3.52 (dt, J = 11.9, 8.3 Hz, 1H), 3.32 (dt, J = 12.5, 6.6 Hz, 2H), 3.06 (dd, J = 14.2, 5.1 Hz, 1H), 3.00 (dd, J = 14.2, 4.6 Hz, 1H), 2.11–1.13 (m, 4H); ^13^C NMR (126 MHz, methanol-d_4_) δ 170.78, 166.96, 157.68, 132.12, 127.62, 116.18, 45.91, 37.66, 29.39, 22.71.

The close agreement between the NMR and HRMS data and the literature confirmed the identity of the two metabolites. The co-production of diolmycin A2, a phenolic polyketide, and maculosin, a diketopiperazine derivative, underscores the chemical diversity of secondary metabolites produced by *Lysinibacillus* sp. JNUCC51. These findings indicate that the strain harbors biosynthetic gene clusters associated with both polyketide synthase (PKS) and nonribosomal peptide synthetase (NRPS) pathways, highlighting its potential as a source of structurally and functionally diverse natural products.

### 3.11. JNUCC 51 Broth Exhibits Skin Compatibility in Human Patch Test

Human skin acts as a vital protective barrier against environmental stressors and various chemical substances present in pharmaceutical and cosmetic products. To ensure product safety and prevent irritation, particularly among sensitive populations such as children, the potential of cosmetic ingredients to induce acute skin irritation must be thoroughly evaluated. In this study, the irritation potential of JNUCC 51 broth (5.0%) was assessed through a human skin patch test.

The test was conducted in accordance with the Personal Care Products Council (PCPC) guidelines and the standard operating procedures of Dermapro Co., Ltd. (Seoul, Republic of Korea). Thirty-two healthy adult women, aged 26–54 years (mean ± SD: 47.78 ± 6.31 years), who met all inclusion and exclusion criteria, participated in the study. For testing, 20 μL of the JNUCC 51 broth samples was applied to a cleansed area on each subject’s upper back and left under occlusion for 24 h. Skin responses were evaluated twice—20 min and 24 h after patch removal—by a board-certified dermatologist according to the PCPC scoring criteria (Table 9). The evaluation results confirmed that JNUCC 51 broth (5.0%) produced no detectable irritation or adverse reactions in any participant, demonstrating excellent skin compatibility and hypoallergenic properties under the test conditions (Table 10).

## 4. Discussion

The isolation and comprehensive characterization of *Lysinibacillus* sp. JNUCC 51 from the high-altitude, oligotrophic volcanic soil of Baengnokdam Crater Lake, Mt. Halla, reveal the untapped biotechnological potential of extremophilic microbiomes inhabiting Jeju Island [1]. This environment—defined by intense ultraviolet exposure, large diurnal thermal fluctuations, and limited nutrient availability—selects for resilient microorganisms with highly adaptive metabolic systems [2,3,7,8,10].

Phylogenetic analysis positioned JNUCC 51 within the genus *Lysinibacillus*, exhibiting 98.52% 16S rRNA similarity with *L. xylanilyticus* DSM 23493ᵀ [16]. However, genome-based indices (ANI 88.76%; dDDH d_6_ = 72.1%, d_0_ = 70.4%) were below the established species delineation thresholds (ANI < 95–96%; dDDH < 70%) [35,36]. These values, consistent with current genomic standards for prokaryotic taxonomy [33,34,37], confirm that JNUCC 51 represents a novel genomic lineage, extending the known taxonomic and ecological diversity of *Lysinibacillus* species [11,12,13,14,15]. Although the dDDH value approaches the species boundary, the substantially lower ANI provides robust evidence of species-level distinctiveness [35,36].

Chemotaxonomic and biochemical characteristics further distinguish JNUCC 51 from its closest relatives. The dominance of iso-C_15:0_ (52.7%) and iso-C_17:0_ (11.6%) fatty acids, the coexistence of MK-6 and MK-7 quinones, and the presence of phosphatidylcholine among the major polar lipids reflect structural adaptations conferring resistance to oxidative and osmotic stress [12,17,18,19]. Such profiles are consistent with *Lysinibacillus* strains inhabiting UV-intensive and nutrient-depleted volcanic environments [1,7,8]. Enhanced growth under acidic (pH 4.0) and moderately saline (up to 5% NaCl) conditions compared with *L. xylanilyticus* further demonstrates adaptive divergence under extreme environmental pressures [16].

The complete genome (5.12 Mb; 37% G + C) encodes 4912 genes, and antiSMASH v8.0 analysis identified ten biosynthetic gene clusters (BGCs), including NRPS, β-lactone, RiPP, terpene, and type III PKS pathways [20,31,32]. This genomic architecture indicates substantial biosynthetic capacity for producing diverse secondary metabolites with potential antimicrobial, antioxidant, and signaling functions [8,9,10]. Bioactivity-guided purification confirmed this capacity through the isolation of diolmycin A2 (a phenolic polyketide) and maculosin (a diketopiperazine), both biosynthetically linked to PKS and NRPS clusters [38,39].

Diolmycin-type metabolites, previously identified in *Bacillus* and *Streptomyces* species, exhibit antimicrobial and anti-inflammatory activities [38], whereas maculosin analogs are known to modulate melanogenesis and oxidative stress in mammalian systems [39]. Their co-occurrence in JNUCC 51 suggests a convergent biosynthetic strategy that enhances ecological competitiveness in volcanic ecosystems [7,8]. Moreover, the predicted terpene and RiPP clusters may produce peptides with moisturizing, antioxidant, or whitening activities, further supporting JNUCC 51 as a valuable source of natural cosmeceutical ingredients [9,10].

Functionally, JNUCC 51 exhibits dual anti-inflammatory and anti-melanogenic properties characteristic of postbiotic candidates [14,15]. The culture supernatant (JNUCC 51B) inhibited nitric oxide production by 24.9% in LPS-stimulated RAW 264.7 macrophages, indicating suppression of the iNOS/NF-κB pathway. Likewise, the ethanol extract and ethyl acetate fraction (JNUCC 51EA) reduced melanin synthesis by 26.7% and tyrosinase activity by 50.6% at 50 µg/mL in α-MSH–induced B16F10 melanocytes, suggesting modulation of the MITF–TYR signaling axis [9]. These results align with previously reported mechanisms of microbial postbiotics that regulate inflammation and pigmentation [14]. Importantly, a human patch test (5% JNUCC 51B; *n* = 32) performed under PCPC guidelines revealed no irritation or sensitization (IRB No. 1-220777-A-N-01-B-DICN25264; approval date: 23 October 2025), confirming dermatological safety and supporting its applicability in topical formulations.

Collectively, these findings identify *Lysinibacillus* sp. JNUCC 51 as a novel volcanic-origin bacterium combining taxonomic novelty, chemotaxonomic distinctiveness, and functional bioactivity [11,12,13,14]. The strain’s BGC diversity, particularly the cryptic NRPS and terpene clusters, suggests additional undiscovered metabolites with antioxidant, surfactant, or microbiome-modulating potential [8,19,20]. Within the broader context of Jeju’s volcanic microbiota, JNUCC 51 complements previously reported isolates such as *Viridibacillus* sp. JNUCC 6 and *Brevibacillus* sp. JNUCC 41 [4,5,6], reinforcing Jeju Island as a promising reservoir for bioactive and cosmeceutical leads [1,7,8].

Future research should focus on elucidating the transcriptional regulation of BGCs, optimizing heterologous expression and fermentation strategies, defining structure–activity relationships of diolmycin A2 and maculosin, and verifying in vivo efficacy using UV-induced pigmentation models [31,32,38,39,40]. Furthermore, establishing formulation stability and scalable production will be essential for translating this strain’s potential into clinically validated, microbiome-friendly skincare solutions Ultimately, genome-guided exploration of extremophilic microorganisms such as *Lysinibacillus* sp. JNUCC 51 provides a sustainable route toward next-generation natural cosmetics and pharmaceuticals [7,8,9,10].

In the context of cosmetic applications, the combined anti-inflammatory, anti-melanogenic, and dermatologically safe properties of JNUCC 51 highlight its strong potential as a multifunctional postbiotic ingredient for next-generation skincare. The activities of diolmycin A2, maculosin, and other predicted BGC-derived metabolites correspond to key functional categories in modern cosmetics, including soothing, brightening, antioxidation, and microbiome-supportive effects. Given the increasing demand for microbiome-friendly and fermentation-derived actives, JNUCC 51 broth and JNUCC 51 extracts represent promising candidates for incorporation into whitening, calming, and anti-inflammatory formulations. However, several limitations remain. The current findings are based primarily on in vitro cell assays, and the molecular mechanisms of the isolated metabolites require further validation. Additionally, long-term skin compatibility, formulation stability, and large-scale production feasibility must be established before commercial translation. Furthermore, to fully resolve the taxonomic position of JNUCC 51, future studies incorporating polyphasic approaches—including experimental comparisons with type strains, chemotaxonomic verification, and laboratory-based DNA–DNA hybridization or its modern equivalents—will be necessary. Despite these constraints, the integrated genomic, chemical, and functional characterization presented in this study positions *Lysinibacillus* sp. JNUCC 51 as a sustainable and scientifically grounded source of novel cosmetic bioactives. Continued investigation, including in vivo efficacy studies and advanced formulation research, will be essential to fully realize its potential in future cosmetic applications. Despite these constraints, the integrated genomic, chemical, and functional characterization presented in this study positions *Lysinibacillus* sp. JNUCC 51 as a sustainable and scientifically grounded source of novel cosmetic bioactives. Continued investigation, including in vivo efficacy studies and advanced formulation research, will be essential to fully realize its potential in future cosmetic applications.

## Figures and Tables

**Table 1:** Growth characteristics of *Lysinibacillus* sp. JNUCC 51 and type strain *L. xylanilyticus* under different environmental conditions.

Characteristic	(a) *Lysinibacillus* sp. JNUCC 51	(b) *L. xylanilyticus*
**pH range**		
pH 4.0	+	–
pH 5.0	+	–
pH 9.0	+	+
pH 10.0	–	–
**NaCl tolerance**		
1%	+	+
3%	+	+
5%	±	–
7%	–	–
10%	–	–
**Temperature tolerance**		
5 °C	–	–
20 °C	–	±
37 °C	+	+
42 °C	+	±
50 °C	–	–

Growth was assessed using OD_600_ after blank subtraction, where (+) indicates OD_600_ ≥ 0.10, (±) 0.02–0.05, and (–) ≤0.01. *Lysinibacillus* sp. JNUCC51 showed broader pH tolerance (pH 4.0–9.0) compared to *L. xylanilyticus*, which grew mainly at pH 5.0–9.0. Under NaCl stress, JNUCC51 sustained growth up to 5%, whereas the reference strain grew only weakly beyond 3%. These results highlight stronger acid tolerance in JNUCC51, with *L. xylanilyticus* being more sensitive to salt.

**Table 2:** Differential API ZYM enzymatic profiles between *Lysinibacillus* sp. JNUCC51 and the type strain *L. xylanilyticus*.

Assay	(a) *Lysinibacillus* sp. JNUCC51	(b) *L. xylanilyticus*	Diagnostic Value
**API ZYM**			
Alkaline phosphatase	–	+	*L. xylanilyticus* specific
Esterase(C4)	+	–	JNUCC 51 specific
Leucine arylamidase	–	+	*L. xylanilyticus* specific
Acid phosphate	+	+	Shared trait
Napthol-AS_BI-phosopatase	+	+	Shared trait
β-galactosidase	–	+	*L. xylanilyticus* specific
α-glucosidase	–	+	*L. xylanilyticus* specific
All other enzyme reactions	–	–	Not differentiating

JNUCC51 exhibited a strain-specific positive reaction for esterase (C4), while the type strain uniquely expressed alkaline phosphatase activity. Both strains were positive for acid phosphatase and naphthol-AS-BI-phosphohydrolase. All other enzyme reactions were negative in both strains and therefore non-differentiating. Note: “+” = positive; “–” = negative.

**Table 3:** Chemotaxonomic comparison of cellular fatty acid profiles between *Lysinibacillus* sp. JNUCC 51 and the type strain *L. xylanilyticus*.

Fatty Acid (%)	(a) *Lysinibacillus* sp. JNUCC 51	(b) *L. xylanilyticus*
**Straight-chain**		
C_14:0_	0.43	ND
C_16:0_	2.09	1.2
**Branched**		
iso-C_11:0_	ND	8.8
anteiso-C_13:0_	0.24	ND
iso-C_14:0_	0.85	2.3
iso-C_15:0_	52.73	50.7
anteiso-C_15:0_	7.16	2.6
iso-C_16:0_	6.93	6.3
iso-C_17__:__0_	11.58	6.2
anteiso-C_17:0_	3.44	1.3
C_15:1_ω5c	2.15	ND
iso-C_17:1_ω10c	2.81	4.0
**Unsaturated**		
C_16:1_ω7c alcohol	4.70	10.4
C_16:1_ω11c	2.50	1.5
**Total branched-chain FA**	~85.74%	~82.2%

Cellular fatty acid compositions of *Lysinibacillus* sp. JNUCC51 and *L. xylanilyticus*. Values are presented as percentages of total fatty acids. ND indicates that the fatty acid was not detected. Fatty acids are grouped into straight-chain, branched-chain, and unsaturated categories.

**Table 4:** Polar Lipid Profile Comparison between *Lysinibacillus* sp. JNUCC 51 and the type strain *L. xylanilyticus*.

Lipid Class	(a) *Lysinibacillus* sp. JNUCC 51	(b) *L. xylanilyticus*	Diagnostic Value
Phosphatidylethanolamine (PE)	+	+	Shared trait
Phosphatidylglycerol (PG)	+	+	Shared trait
Diphosphatidylglycerol (DPG)	+	+	Shared trait
Phosphatidylcholine (PC)	+	−	JNUCC 51 specific
Unidentified phospholipid (PL)	+	−	JNUCC 51 specific
Unidentified lipid (L)	+	−	JNUCC 51 specific

Polar lipid analysis of strain JNUCC 51 revealed six components—phosphatidylethanolamine (PE), phosphatidylglycerol (PG), diphosphatidylglycerol (DPG), phosphatidylcholine (PC), an unidentified phospholipid (PL), and an unidentified lipid (L). In contrast, the type strain of *L. xylanilyticus* possessed three major polar lipids (DPG, PG, and PE). Results are based on TLC with molybdophosphoric acid and ninhydrin staining.

**Table 5:** General Genomic Features of *Lysinibacillus* sp. Strain JNUCC 51.

*Lysinibacillus* sp. Strain JNUCC 51	
Genome size (bp)	5,122,675
Total number of contigs	1
Contigs N50 (bp)	5,122,675
Plasmid	0
G + C content (%)	37
Genome coverage	221×
Number of chromosomes	1
Total number of predicted genes	4912
Total number of protein-coding genes	4615
Total number of pseudogenes	141
Total number of tRNA-coding genes	108
Total number of rRNA-coding genes (5S, 16S, 23S)	15, 14, 14
Total number of ncRNA-coding genes	5

**Table 6:** Digital DNA–DNA hybridization (dDDH) values between *Lysinibacillus* sp. JNUCC 51 and related *Lysinibacillus* species.

Subject Strain	dDDH (d_0_, in %)	C.I. (d_0_, in %)	dDDH (d_4_, in %)	C.I. (d_4_, in %)	dDDH (d_6_, in %)	C.I. (d_6_, in %)	G + C Content Difference (in %)
*Lysinibacillus xylanilyticus* DSM 23493	70.4	[66.5–74.1]	67	[64.0–69.8]	72.1	[68.6–75.3]	0.25
*Lysinibacillus agricola* FJAT-51161	45	[41.6–48.4]	41.7	[39.2–44.3]	43.9	[40.9–46.9]	0.13
*Bacillus decisifrondis* DSM 17725	47.4	[44.0–50.8]	41.4	[38.9–44.0]	45.9	[42.9–48.9]	0.27
*Lysinibacillus zambalensis* M3	55.2	[51.7–58.7]	41.3	[38.8–43.8]	52.4	[49.3–55.4]	0.38
*Lysinibacillus pakistanensis* JCM 18776	27.5	[24.2–31.1]	29	[26.6–31.5]	26.5	[23.7–29.7]	0.54
*Lysinibacillus parviboronicapiens* BAM-582	23.2	[24.2–31.1]	26.7	[24.4–29.2]	22.6	[19.8–25.7]	0.67
*Lysinibacillus irui* IRB4-01	24.8	[21.5–28.4]	26.6	[24.2–29.0]	23.9	[21.1–27.0]	0.52
*Lysinibacillus macroides* DSM 54	20.6	[17.4–24.3]	26.1	[23.8–28.6]	20.3	[17.6–23.4]	1
*Lysinibacillus sphaericus* KCTC 3346	22.4	[19.1–26.0]	26.1	[23.7–28.6]	21.8	[19.0–24.9]	0.24
*Lysinibacillus mangiferihumi* M-GX18	21.5	[18.2–25.1]	26	[23.7–28.5]	21	[18.3–24.1]	0.21
*Lysinibacillus tabacifolii* K3514	22.2	[19.0–25.9]	25.8	[23.4–28.3]	21.7	[18.9–24.8]	0.27
*Lysinibacillus capsici* PB300T	24.7	[21.4–28.3]	25.7	[23.4–28.2]	23.7	[20.9–26.8]	0.64
*Lysinibacillus boronitolerans* NBRC 103108	24.7	[21.4–28.4]	25.5	[23.2–28.0]	23.7	[20.9–26.8]	0.68
*Lysinibacillus pinottii* PB211	24.5	[21.2–28.2]	25.5	[23.2–28.0]	23.5	[20.7–26.6]	0.25

Values represent percent relatedness calculated by GGDC 4.0 using formulas d_0_, d_4_, and d_6_, with 95% confidence intervals. G + C content differences indicate compositional divergence.

**Table 7:** OrthoANIu results between *Lysinibacillus* sp. JNUCC 51 (A) and *L. xylanilyticus* (B).

Title 1	Title 2
OrthoANIu value (%)	88.76%
Genome A length (bp)	5,122,440
Genome B length (bp)	5,049,000
Average aligned length (bp)	2,315,154
Genome A coverage (%)	45.20
Genome B coverage (%)	45.85

**Table 8:** Biosynthetic Gene Clusters (BGCs) predicted in the genome of strain *Lysinibacillus* sp. Strain JNUCC 51 by antiSMASH.

Region	Type	From	To	Similarity Confidence	Most Similar Known Cluster	BGC Class
1	NI-siderophore	342,425	373,986	Low	Petrobactin	Other:other
2	Terpene	657,484	678,305			
3	Terpene	782,764	803,387			
4	Betalactone	1,863,328	1,887,790	Low	Fengycin	NRPS:Type I
5	NRPS	2,109,436	2,161,607			
6	Azole-containing-RiPP	2,240,880	2,268,297			
7	T3PKS	2,748,877	2,789,959	Low	Bacillibactin/E/F	NRPS:Type I
8	Terpene-precursor	2,846,049	2,866,951			
9	NRPS	3,131,122	3,195,262			
10	Terpene-precursor	4,342,547	4,363,428			

A total of 10 biosynthetic gene clusters (BGCs) were identified in the genome of *Lysinibacillus* sp. JNUCC 51 by antiSMASH. These include NRPS, terpene, RiPP, and betalactone types. Notably, Clusters 5 and 9 were predicted as Type I NRPSs, partially resembling *Fengycin* and *Bacillibactin*, while some terpene-related clusters lacked known reference matches.

**Table 9:** Grading system for the primary skin irritation test.

Grade	Description of Clinical Observations
**+1**	Slight erythema
**+2**	Moderate erythema, possibly with barely perceptible edema at the margin, papules may be present.
**+3**	Moderate erythema, with generalized edema
**+4**	Severe erythema with severe edema, with or without vesicles
**+5**	The severe reaction spread beyond the area of the patch

**Table 10:** Results of the human skin primary irritation test (*n* = 32).

No.	Test Sample	No. of Responder	1st Assessment				2nd Assessment				Reaction Grade (R) *
			+1	+2	+3	+4	+1	+2	+3	+4	
**1**	JNUCC 51 broth (5.0%)	0	0	0	0	0	0	0	0	0	0

* None to slight: 0.00 ≤ R < 0.87.

## Data Availability

The original contributions presented in this study are included in the article/Supplementary Materials. Further inquiries can be directed to the corresponding author.

## Supplementary Materials

The following supporting information can be downloaded at: https://www.mdpi.com/article/10.3390/microorganisms13122786/s1, Figure S1: ^1^H NMR of the Diolmycins A2; Figure S2: ^13^C NMR of the Diolmycins A2; Figure S3: HRMS of the Diolmycins A2; Figure S4: ^1^H NMR of the maculosin; Figure S5: ^13^C NMR of the maculosin.
