# Supplementary material for: Taxonomic Characterization, Whole-Genome Sequencing, and Cosmetic Potential of Lysinibacillus sp. JNUCC 51 Isolated from Baengnokdam Crater Lake, Mt. Halla
Source: Microorganisms. 2025 Dec 7;13(12):2786. doi: 10.3390/microorganisms13122786 (PMC12735944; doi:10.3390/microorganisms13122786)

Taxonomic Characterization, Whole-Genome Sequencing, and Cosmetic Potential of *Lysinibacillus* sp. JNUCC 51 Isolated from Baengnokdam Crater Lake, Mt. Halla

Ji-Hyun Kim [1], Xuhui Liang [1], Mi-Na Kim [1] and Chang-Gu Hyun [1,*]

[1]Department of Chemistry and Cosmetics, Jeju Inside Agency and Cosmetic Science Center, Jeju National University, Jeju 63243, Republic of Korea
*Correspondence: cghyun@jejunu.ac.kr; Tel.: +82-64-754-1803

Contents

Figure S1. ^{1}H NMR of the Diolmycins A2..2

Figure S2. ^{13}C NMR of the Diolmycins A2...2

Figure S3. HRMS of the Diolmycins A2...3

Figure S4. ^{1}H NMR of the maculosin...3

Figure S5. ^{13}C NMR of the maculosin 4

Figure S1. ^{1}H NMR of the Diolmycins A2

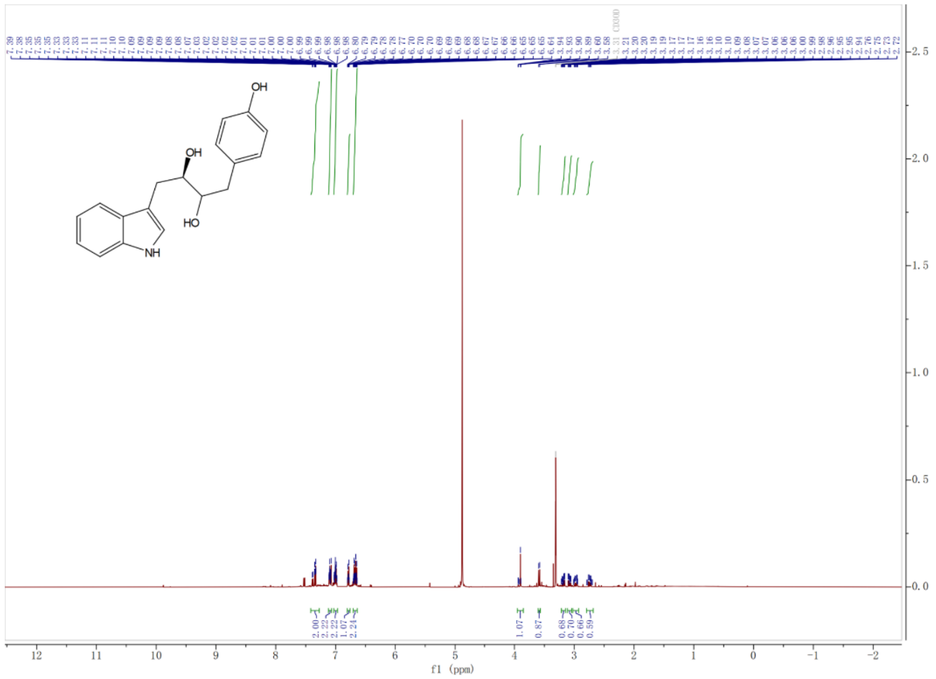

Figure S2. ^{13}C NMR of the Diolmycins A2

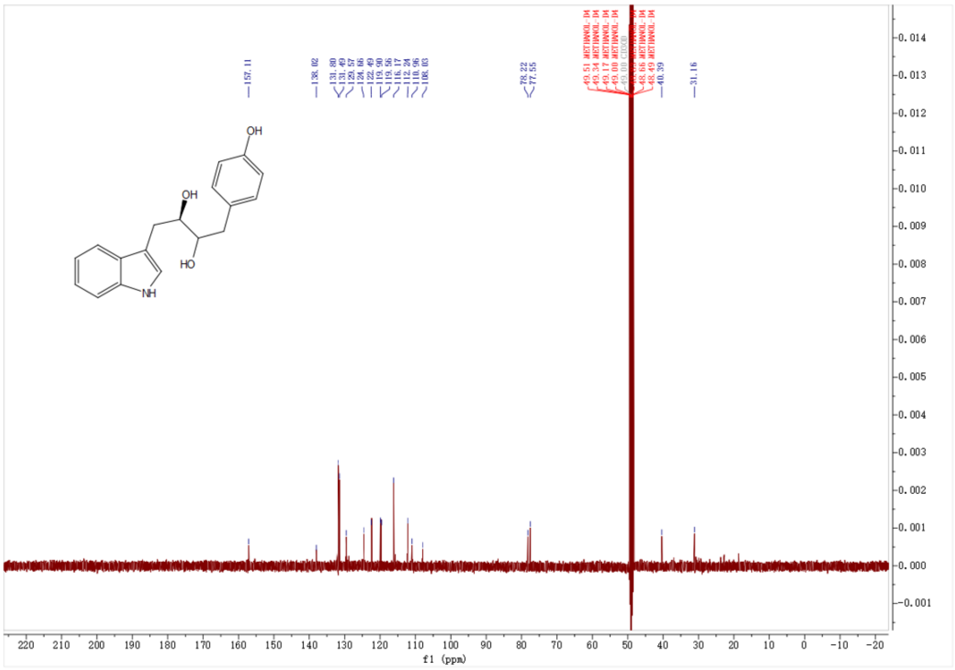

Figure S3. HRMS of the Diolmycins A2

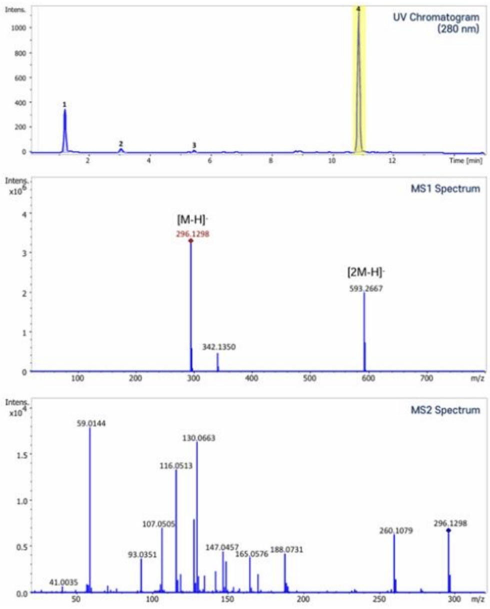

Figure S4. ^{1}H NMR of the maculosin

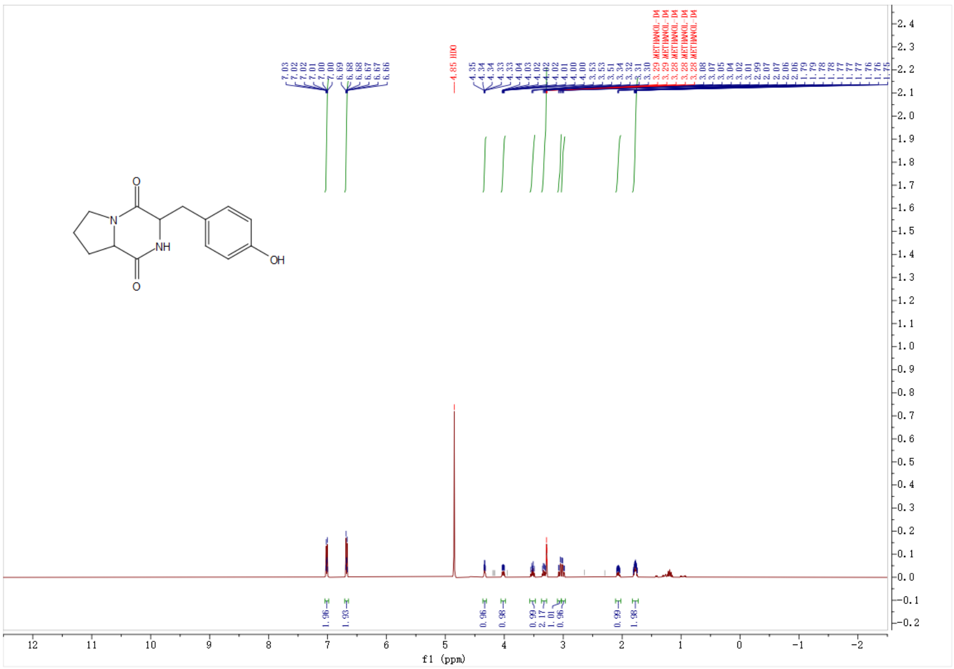

Figure S5. ^{13}C NMR of the maculosin

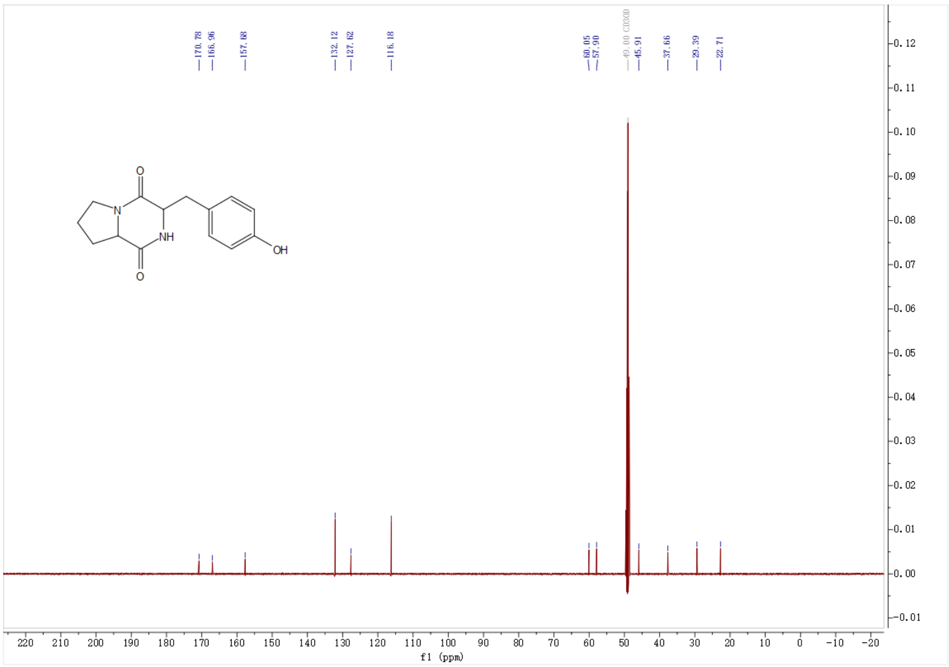

Supplement: Supplementary file 1 [file microorganisms-13-02786-s001.zip › microorganisms-4003655-supplementary.pdf]
